# Effectiveness of a Global Multidisciplinary Supportive and Educational Intervention in Thermal Resort on Anthropometric and Biological Parameters, and the Disease-Free Survival after Breast Cancer Treatment Completion (PACThe)

**DOI:** 10.1155/2020/4181850

**Published:** 2020-05-05

**Authors:** Marie-Paule Vasson, Fabrice Kwiatkowski, Adrien Rossary, Sylvie Jouvency, Marie-Ange Mouret-Reynier, Martine Duclos, Isabelle Van Praagh-Doreau, Armelle Travade, Yves-Jean Bignon

**Affiliations:** ^1^Jean Perrin Comprehensive Cancer Centre, Department of Nutrition, 58 Rue Montalembert, 63011 Clermont-Ferrand, France; ^2^University of Clermont Auvergne, INRA, UMR 1019 Human Nutrition Unit, CRNH-Auvergne, 28 Place Henri Dunant, 63000 Clermont-Ferrand, France; ^3^Jean Perrin Comprehensive Cancer Centre, Department of Oncogenetics, 58 Rue Montalembert, 63011 Clermont-Ferrand, France; ^4^University of Clermont-Auvergne, Laboratory of Mathematics, Probabilities and Applied Statistics, 28 Place Henri Dunant, 63000 Clermont-Ferrand, France; ^5^Jean Perrin Comprehensive Cancer Centre, Department of Oncology, 58 Rue Montalembert, 63011 Clermont-Ferrand, France; ^6^Gabriel Montpied University Hospital, Department of Sport Medicine and Functional Explorations, 58 Rue Montalembert, 63000 Clermont-Ferrand, France; ^7^Centre République, Department of Senology, 99 Avenue de La Republique, 63100 Clermont Ferrand, France

## Abstract

A growing knowledge highlights the strong benefit of regular physical activity in the management of breast cancer patients, but few studies have considered biological parameters in their outcomes. In the prospective randomised trial after breast cancer treatment completion “PACThe,” we determined the effects of physical activity and nutritional intervention on the biological and anthropometric status of patients after one year of follow-up, and clarified the link between biomarkers at allocation and disease-free survival. 113 patients from the population of the “PACThe” study (*n* = 251) were analysed for biological parameters. Patients were randomized after chemotherapy in two arms: the intervention “SPA” receiving a 2-week session of physical training, dietary education, and physiotherapy (*n* = 57), and the control “CTR” (*n* = 56). Diet questionnaire, anthropometric measures, and blood parameters were determined at allocation and one year later. Survival and recurrence were checked over 7 years. Data were considered as a function of BMI, i.e., ≤25 for normal, 25–30 for overweight, and >30 for obese patients. At allocation, the large standard deviation for nutrient-intake values reflected an unbalanced diet for some patients in the three groups. At one-year follow-up, we noticed an increase in glucose (*p* < 10^−6^), insulin (*p* < 10^−7^), and adiponectin (*p* < 0.022) plasma levels for both intervention arms, which were more accentuated for the >30 groups. Using the Cox model, we demonstrated that the highest testosterone plasma values were linked to an increase of the recurrence risk (HR [CI–95%] = 5.06 [1.66–15.41]; *p*=0.004). One-year after a global multidisciplinary supportive and educational intervention, we found few anthropometric and biological changes, mainly related to the patient's initial BMI. We highlighted the importance of plasma testosterone in the evaluation of patient's recurrence risk. Future studies would help better understand the mechanisms by which such multidisciplinary interventions could interact with breast cancer recurrence and define the most effective modalities.

## 1. Introduction

Over many years, growing knowledge has indicated the strong benefit of regular physical activity in the management of breast cancer patients [1]. Despite an extensive literature of clinical trials, data from these studies showed positive but modest effects, which may be underestimated due to great variability in the intervention strategies and intensity of monitoring [2, 3]. These interventions produce short-term changes in physical activity and patient behaviour, but data are scarce on recurrence and long-term follow-up. Some studies have highlighted long-term barriers to exercise after diagnosis of breast cancer, including psychological barriers (e.g., low motivation and dislike of gym), environmental barriers (e.g., employment priority and low access to facilities), and lack of time [4]. Regarding the large variability of practice procedures, further research is required to investigate how to sustain positive effects of exercise over time and to determine essential attributes of exercise (mode, intensity, frequency, duration, and timing) by cancer type and cancer treatment for optimal effects [5]. The introduction of wearable activity monitors into cancer care could improve the understanding of the association between physical activity and patient behaviour, as previously suggested [1].

Moreover, analyses are needed to provide insight into how physical activity interventions work. Such studies should accelerate the identification of effective behaviour changes and permit the development of evidence-based practice with better standardisation. Currently, the mechanisms by which physical activity mediates its benefits remain unclear [6]. Most hypotheses regarding the biological pathways have focused on the impact of obesity on breast cancer risk and recurrence. In that field, the main research axes are, first, the implication of sex hormones, including both oestrogens and androgens (testosterone) [7]; second, the implication of metabolic hormones, such as insulin/insulin-like growth factor (IGF) axis and adipocytokines (leptin and adiponectin) [8]; and third, the implication of inflammatory factors (C reactive protein, CRP) [9]. None of these axes has clearly demonstrated efficiency in clinical trials, despite evidence of increased quality of life (QoL), reduced body weight in obese patients, and reduced recurrence.

The majority of studies that investigate the benefits of physical activity and nutritional interventions in breast cancer focus on weight loss, cardiorespiratory capacity, QoL, and overall well-being [5, 10, 11], but few of them considered the biological parameters of the patients in their outcomes [12, 13].

Taking into account these data and the interactions between physical activity and BMI, we performed a prospective randomized trial “Programme of Accompanying women after breast Cancer treatment completion in Thermal resorts” (PACThe) for complete-responder breast cancer patients after chemotherapy. In this trial, we demonstrated that the 2-week intervention durably influences the QoL of breast cancer patients after both short-term [14] and long-term treatment [15]. In the present study, we determined the effects of PACThe intervention on the biological and anthropometric status of patients after one-year follow-up and the link between the biomarkers and disease-free survival with seven years of follow-up after completion of breast cancer treatment.

## 2. Patients and Methods

### 2.1. Participants

Two hundred and fifty-one nonmetastatic breast cancer patients were enrolled between 2008 and 2010, as previously described [14]. The main inclusion criteria were notably invasive nonmetastatic breast carcinoma; less than 9 months after chemotherapy/radiotherapy completion, complete remission, 18.5 < BMI < 40 kg/m^2,^ and written informed consent. Half of the 251 patients (*n* = 113) were investigated for biological parameters in the present study.

### 2.2. Study Design

Patients were randomized into two groups: “SPA,” for the group attending the 2-week session in thermal centres, and “CTR,” for the control group. The 2-week session performed in thermal centres included consultations with physicians, nutritionists, and psycho-oncologists; physical activity supervised by a physiotherapist for 2 h daily with endurance activities, strength training, and flexibility/stretching; SPA care consisting of bath, shower, and massage for half an hour per day; aesthetic care; and dietary meals with adapted menus, dietary education, and caloric intake limited to 1700–2000 kcal/day.

Besides standard oncological follow-up of the patients in the two groups, personal consultations with a dietician were organized to perform anthropometric measurements, provide dietary advice, and give encouragement for daily physical activity. Evaluation of survival/recurrence was made by patients' oncologist, with a follow-up period of 7 years [14]. The overall protocol design is available in a supplementary file.

### 2.3. Data Collection

Before randomization and at one year, the following analyses were performed on half of the population (SPA: *n* = 57; CTR: *n* = 56):

1. Diet questionnaire
2. Dietitians evaluated oral intake based on a 72-h self-reported diet questionnaire.
3. Body composition
4. Body weight was measured at each personal consultation. Lean body mass (LBM), fat mass (FM), and total body water were evaluated by multifrequency bioelectrical impedance analysis (Bodystat Quadscan 4000) using 5, 50, 100, and 200 kHz. Tricipital skin-fold thickness was measured using a skin-fold caliper (Harpenden caliper). To assess central fat distribution, the waist circumference (WC) was evaluated to the nearest 0.5 cm using a standard tape measure placed between the lowest rib and the iliac crest, with the patient in the standing position. The hip circumference (HC) was estimated using a standard tape measure placed horizontally at the widest point on the hip.
5. Blood sampling and biological assays
6. Blood samples were collected at allocation and at one year. Plasma levels of biomarkers were determined as follows: glucose and HDL-cholesterol (colorimetry methods), C-reactive protein, and transthyretin (immunonephelometry) were determined at the biomedical laboratory of the recruiting centre; insulin and testosterone (ELISA) were determined at the hospital biochemistry laboratory (Clermont-Ferrand); IGF-1, leptin, and adiponectin (luminex) were determined at the Genotool platform (Toulouse); and CA 15-3 was determined at the anticancer centre radiobiology laboratory (Clermont-Ferrand).
7. Recurrence follow-up
8. Disease-free interval was computed as months elapsed from date of randomization to documented breast cancer recurrence during seven years after breast cancer treatment completion. All recurrence types were considered, either local or distant (nodes, metastatis, and/or contralateral breast cancer).

### 2.4. Statistical Considerations

Protocol design consisted of a multicentre parallel randomized prospective trial. Data were analysed using the intention-to-treat principle. Descriptive statistics are presented with mean ± standard deviation (SD) for Gaussian quantitative variables. Outcomes are shown with 95% confidence intervals. Categorical variables are described using counts by class and frequencies (%).

Comparison of outcomes per allocation group and per BMI class was tested with Student's *t*-test, one-way analysis of variance (ANOVA), or the Kruskal-Wallis H-test depending on homoscedasticity or normality of distributions. Two-way ANOVA was used to compare longitudinal variations between allocation groups, but without an interaction test because of unequal class sizes. Categorical data were compared with chi^2^ test. To test the association between two quantitative parameters, Pearson's correlation coefficient was used, or Spearman's rank correlation if distributions were not Gaussian. Survival curves were drawn using Kaplan-Meier's method, and comparison of curves was performed using the Log-rank test. A backward and stepwise Cox proportional hazard regression model was used to perform the multivariate analysis of survival. Cutoff values of biological parameters to draw survival curves were chosen among quartiles of distribution.

All tests were two-sided and the nominal level of significance was 5%. Randomisation and statistics were performed using SEM software [16].

## 3. Results

**Figure 1:**
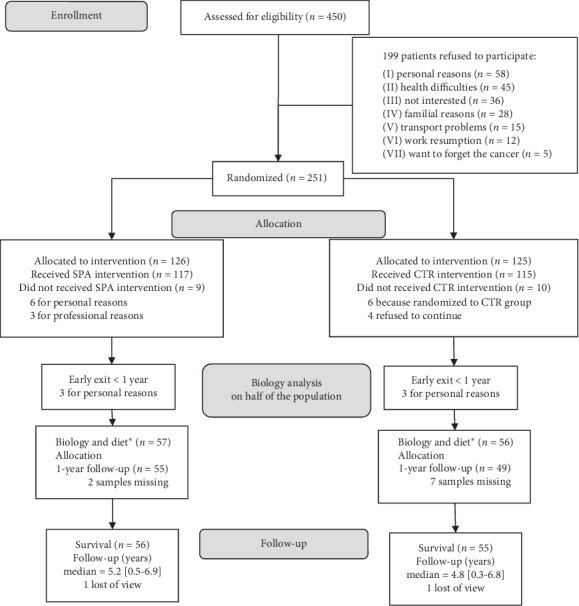
Allocation diagram and flow chart. ^*∗*^Diet, nutritional, and body data collection.

Biological parameters were evaluated at allocation for half of the 251 patients: *n* = 57 for the “SPA” experimental group and *n* = 56 for the “CTR” control group (Figure 1). These 113 patients are referred to hereafter as the biological study population. At one year post-inclusion, 13 patients withdrew for familial or professional reasons, and 53 and 47 patients remained, respectively, for the SPA and CTR groups. The main covariates were distributed similarly between the allocation groups (Table 1). Cancer treatments were similar and standard for invasive tumours. Most patients' tumours were HR positive and treated using hormonotherapy, and a few (Her2+ tumours) using targeted therapy.

### 3.1. Diet, Body, and Biological Parameters at Allocation

Results of the biological study population were considered in function of BMI scale and divided into three subgroups, i.e., ≤25 kg/m^2^ for normal BMI, [25–30 kg/m^2^] for overweight, and >30 for obesity (Tables 2 & 3). Overall diet mean results (Table 2) were within adult nutritional recommendations (17.3% ± 4.1, 46.7% ± 10.4, and 35.5% ± 8.6, respectively, for protein, carbohydrate, and lipid intakes). A large dispersion of values was observed, resulting in no significant difference between BMI subgroups except for total energy intake (TEI) (*p*=0.038) and lipid intake in gram/day (*p*=0.034). The large standard deviation for each nutrient-intake value reflected an unbalance diet for some patients in the three BMI subgroups.

All body parameters (Table 2) differed significantly by BMI subgroup (*p* < 10 − 7). As expected, the lean mass/fat mass ratio decreased with the BMI due to the expansion of the body fat mass, i.e., 2.4 ± 0.6, 1.7 ± 0.3, and 1.3 ± 0.3, respectively, for normal, overweight, and obese subgroups (*p* < 10 − 7).

As previously noticed, we observed a large dispersion of all biological parameter values (Table 3) regardless of BMI subgroup. Increased plasma levels of CRP (*p* < 10 − 5), insulin (*p* < 10 − 4), and leptin (*p* < 10 − 7) showed dysmetabolic disorders associated with overweight/obesity. As expected, the ratio of leptin/adiponectin significantly increased with BMI (0.53 ± 0.51, 1.26 ± 1.28, and 3.23 ± 3.86, respectively, for normal, overweight, and obese groups, *p* < 10 − 7). Conversely, a significant decrease in HDL-C level with BMI (*p* < 10 − 4) was observed. Transthyretin, similar between groups, was in the physiological range, showing no malnutrition disorders in the studied population. Other parameters (glucose, IGF-1, testosterone, and CA 15-3) were in the normal range, with no difference between BMI groups except for CA 15-3 (*p*=0.014).

### 3.2. Changes in Diet, Body, and Biological Parameters One Year Later

One year after inclusion, Diet consumption, body, and biological parameters of patients were reevaluated one year after inclusion. All the raw data are presented by BMI subgroups in two supplementary data files: one for the SPA group (Supplementary Table 1) and one for the CTR group (Supplementary Table 2). Variations in each parameter between inclusion and one-year follow-up are shown in Tables 4 and 5 and analyzed according to the intervention group (SPA effect), one-year follow-up (time effect), and BMI subgroups (BMI effect).

No significant difference was observed for diet parameters (Table 4) regardless of the intervention group, the time window, or the BMI subgroup, except for the total energy intake with time (*p*=0.039). For the SPA group, total energy intake remained stable for BMI subgroups ≤25 and [25–30 kg/m^2^], whereas a strong reduction (−400 kcal/d) in the BMI >30 subgroup led to both carbohydrate (−21.5%) and lipid (−13.8%) intake decreases without change in patients' weight. For the CTR group, total energy intake decreased for ≤25 and >30 BMI subgroups due to a reduction in protein, carbohydrate, and lipid intakes. However, an increase in the mean body weight of 1 kg was observed for each BMI subgroup (supplementary data), which was not significant because of the large dispersion of individual values.

For body parameters (Table 4), we observed that only the BMI effect was significant (*p* < 10^−7^). All the parameters were significantly related to BMI but remained stable considering both SPA and time effects. For the SPA and CTR >30 BMI subgroups, a reduction in brachial and abdominal circumferences tended to correlate with an increase in hip circumference.

No significant SPA effect was observed for biological parameters (Table 5), except for transthyretin (*p*=0.041) and CA 15-3 (*p*=0.04) plasma levels, although these remained in the normal ranges. For the time effect, a significant increase in both glucose (*p*=0.04) and insulin (*p*=0.035) and a decrease in HDL-C (*p*=0.027) plasma levels were observed. As expected, several parameter variations were related to BMI in the two groups as previously shown at allocation. Notably, we noticed an increase in glucose (*p* < 10 − 6), insulin (*p* < 10 − 7), and adiponectin (*p*=0.022) plasma levels regardless of the intervention group and more accentuated plasma levels for the >30 BMI subgroups. Conversely, a decrease in HDL-C plasma levels was observed (*p*=0.007).

We found significant positive correlations in the biological study population between leptin/adiponectin ratio and insulin (r = 0.46, *p* < 10 − 7) and CRP (*r* = 0.46, *p* < 10 − 7) and a negative correlation with HDL-C (*p*=−0.46, *p* < 10 − 7). The leptin/adiponectin ratio was strongly correlated with waist circumference (*r* = 0.67, *p* < 10 − 7), BMI (*r* = 0.51, *p* < 10 − 7), and cell mass (*r* = 0.46, *p* < 10 − 7). Moreover, despite the absence of variation in testosterone plasma level with SPA, time, or BMI effects, this parameter was significantly associated (i) positively with body weight (*r* = +0.15, *p*=0.03), cell mass (*r* = +0.19, *p*=0.0072), arm circumference (*r* = +0.15, *p*=0.026), WC/HC ratio (*r* = +0.15, *p*=0.027), and transthyretin (*r* = +0.15, *p*=0.028) and (ii) negatively with TEI (*r* = −0.16, *p*=0.022) and HDL-C (*r* = −0.19, *p*=0.007).

### 3.3. Biological Parameters and Recurrence Relation

We tested the association between biomarker plasma levels at allocation expressed in quartiles and the risk of recurrence during the seven-year follow-up. Highest HDL-cholesterol values were associated with the best survival without recurrence (*p*=0.047). Conversely, the lowest testosterone and CA 15-3 values were associated with longer disease-free survival (*p*=0.001 and 0.03, respectively) (Table 6).

**Figure 2:**
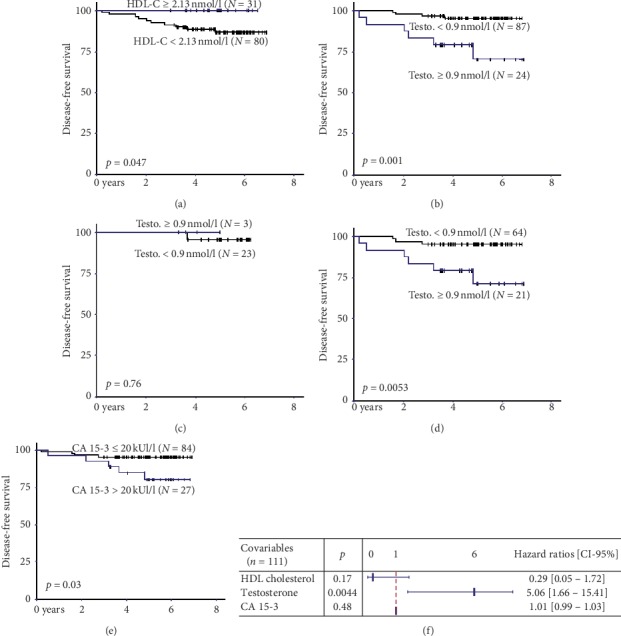
Survival curves and hazard ratios for HDL-cholesterol, testosterone, and CA 15-3. (a) HDL-cholesterol. (b) Testosterone—all patients. (c) Testosterone—patients without hormonotherapy. (d) Testosterone—patients with hormonotherapy. (e) CA 15-3. (f) Hazard ratios (Cox model). Threshold values for HDL-cholesterol, testosterone, and CA 15-3 at allocation correspond to the 75% percentile values. They were used to draw survival curves using Kaplan-Meier's method. Comparison of curves was performed using the Log-rank test. Backward stepwise Cox proportional hazard regression model was used to perform the multivariate analysis of survival. All tests were two-sided, and the nominal level of significance was 5%.

The survival curves for these three biomarkers were done in function of the calculated significant threshold values (2.13 mmol/l, 0.9 nmol/l, and 20 kUI/l, respectively, for HDL-C, testosterone, and CA 15-3) (Figures 2(a), 2(b), 2(e)). For testosterone, two other survival curves were plotted taking into account the hormonotherapy status of patients (Figures 2(c), 2(d)). These latter showed that testosterone was relevant for disease-free survival only in patients treated with hormonotherapy (*p*=0.012 vs. *p*=0.69, respectively, for patients with and without hormonotherapy). Using the Cox model, the link between these variables and disease-free survival was tested and demonstrated that only the highest testosterone values predicted increased recurrence risk (HR [CI–95%] = 5.06 [1.66–15.41], *p*=0.004) (Figure 2(f)).

## 4. Discussion

In the present study, we determined the effects of PACThe intervention (i.e., medical, nutritional, and psychological monitoring; physical activity training; SPA; and aesthetic care) on the biological and anthropometric status of patients at allocation and after one-year follow-up.

As obesity has an impact on biological status and is a risk factor for breast cancer, we chose to discuss the data according to three BMI subgroups defined as follows: ≤25 kg/m^2^ for normal BMI, [25–30 kg/m^2^] for overweight, and >30 for obesity. At allocation, the study population's repartition into BMI subgroups was similar to that of the same-age female French population, as previously described [17]. The diet intakes are in accordance with the adult nutritional recommendations for all groups. We noted no difference between the three subgroups but a great variation in declared intakes, particularly in the obese group, raising doubts as to the reliability of the consumption-data collection based on a 72-h self-report.

At allocation, after the completion of breast cancer treatment, the biological and body parameters of the population were in accordance with the usual observed values for normal, overweight, and obesity status. Considering the mean value for each parameter defined as EGIR metabolic syndrome criteria (glucose > 6.1 mmol/l, HDL-C < 1 mmol/l, insulin >18 mUI/l (QR4), and waist circumference > 80 cm), neither overweight nor obesity subgroups met the three required criteria [18]. Among these parameters, only the central criterion of obesity (waist circumference) was above the limit value and emerged as the earliest criterion of metabolic syndrome under our conditions. However, considering the large value dispersion of all these parameters, some patients of both overweight and obese groups could present a metabolic syndrome.

Obesity is well-known to be associated with elevated circulating levels of insulin, insulin-like growth factor 1 (IGF-1), leptin, and inflammation [19]. In our study, we observed a significant increase in CRP, insulin, leptin plasma levels, and the ratio leptin/adiponectin in parallel with significantly increased adiposity markers (fat mass, arm, waist, and hip circumferences). As expected, circulating anti-inflammatory adiponectin was decreased, reinforcing the sub-chronic inflammation associated with obesity and related to the risk of recurrence [20]. Surprisingly, no difference was observed for IGF-1 and testosterone plasma contents, contrary to previous observations [8, 13], probably due to the huge variability of individual values. Their plasma concentrations were maintained in the physiological range for the female population of corresponding age [21, 22].

Globally, as measurements were performed after completion of breast cancer treatment, body and biological parameters seemed to be more linked to BMI status than to breast disease. Nevertheless, as previously described [23–25], we cannot exclude that the breast cancer therapy may be another cause of metabolic disturbances at allocation. That may be the reason for the great variability observed for all parameters regardless of the BMI subgroup.

One year after inclusion, the impact of the SPA intervention on diet, body, and biological parameters was evaluated. Only transthyretin and CA 15-3 plasma levels were significantly affected by the SPA intervention. Transthyretin, one of the thyroid hormone carriers, is recognized as an acute malnutrition marker whose hepatic synthesis is reduced in case of inflammation [26]. In our study, transthyretin levels remained in the normal range and seemed to be without biological meaning in regard of their tiny variations and the absence of inflammation and of lean mass changes. Breast cancer is generally not associated with malnutrition or sarcopenia, especially so long after treatment [27]. CA 15-3 is frequently used for diagnosis and follow-up of breast cancer [28]. In our study, an *a posteriori* bias appeared for these biomarker data because the CTR group patients presented higher CA 15-3 concentrations than the SPA group at allocation (Supplementary Table 2). One year after treatment completion, as none of the patients was in recurrence, CA 15-3 values decreased under the threshold of 30 kU/l, confirming the efficacy of the therapy [29, 30]. In accordance with previous studies showing modest effects on body and biological parameters of physical activity and nutritional interventions [31, 32], our study shows the lack of one-year impact of a 2-week SPA intervention.

Some metabolic disorder changes were pointed out at one-year follow-up (time effect). Despite a decrease in total energy intake, patients presented an increase in glucose and insulin plasma levels associated with a decrease in HDL-C. These parameters suggest the development of insulin resistance independently of the BMI effect for overweight patients and the reinforcement of insulin resistance for obese patients. These observations are in agreement with previous studies which considered breast cancer as a metabolic disease, with insulin resistance, sub-chronic inflammation, and dysmetabolism induced by therapy [33, 34]. Moreover, an increased risk for metabolic syndrome and obesity has been described in long-term breast cancer survivors [35].

If women with breast cancer frequently lose weight during chemotherapy, a common unwanted long-term effect of this therapy is weight gain, which often ranges 2–6 kg [10, 36] and penalizes mainly patients with adjuvant therapy [37]. In our study, weight gain was modest (less than 1 kg) and concerned mainly the overweight BMI groups, of whom the majority were under hormonal adjuvant therapy. Thus, weight control and diet intervention are important to improve care and control of recurrence risk in posttreatment breast cancer patients [38]. In our study, the reduction in the total energy intake provided by diet modification, especially carbohydrate and lipid intakes, demonstrated the efficacy of patient's nutritional information.

As described at allocation, BMI was the major factor conditioning body and biological parameter changes one year later. For body parameters, we noted high central adiposity (waist and hip circumferences) in the overweight and obese groups. The same biomarker variations were observed and reinforced for the overweight and obese subgroups (i.e., increase in insulin, leptin, and CRP, and decrease in HDL-C). Moreover, these metabolic disorders induced an increased glycaemia and a decreased adiponectinemia in relation to more pronounced insulin resistance and sub-chronic inflammation [20]. Thus, the obese groups presented two EGIR criteria for metabolic syndrome (glucose and waist circumference) one year after breast cancer treatment completion. This confirms previous studies establishing that breast cancer posttreatment increases the risk of metabolic syndrome [39, 40].

Finally, we clarified the link between biological markers at allocation and disease-free survival over seven years of follow-up after breast cancer treatment completion. We confirmed the interest of three biomarkers commonly used in the determination of recurrence risk: the highest plasma values of HDL-C and the lowest plasma values of testosterone and CA 15-3 were associated with a reduced risk of recurrence [41–43]. HDL-C is linked to metabolic disorders and is often related to androgen metabolism [44]. Cholesterol is clearly demonstrated to be a key regulator of breast cancer tumours [45]. Favouring liver cholesterol clearance, an increase in HDL-C limits the availability of cholesterol for recurrent cancer stem cells [46]. In our study, patients with the highest circulating HDL-C presented the lowest recurrence risk. However, this protective effect was not retrieved in the multivariate Cox model, limiting the interest of circulating HDL-C determination in recurrence monitoring.

As previously noted, CA 15-3 is a useful marker for breast cancer follow-up: the circulating value is directly related to the stage and mass of the tumour [29]. In our study, although the lowest circulating CA 15-3 values were associated with the lowest recurrence risk, the multivariate Cox model did not confirm this observation. This is in agreement with the literature, which has established the interest in CA 15-3 for monitoring breast tumour growth, but its poor prognostic value for recurrence risk [28, 30].

In our study, only testosterone presented a significant hazard ratio with disease-free survival; that is, the highest circulating values (>0.9 nmol/l) were associated with recurrence risk multiplied by ≈5 (HR = 5.06 [1.66–15.41]). Notably, this link between testosterone and recurrence risk only applied to patients receiving adjuvant hormonotherapy. This observation confirms Venturelli's observation of increased recurrence risk for testosterone plasma concentration above 0.96 nmol/l with a hazard ratio of 4.68 for overweight women but not for obese ones [47]. Testosterone is strongly associated with the androgen hypothesis of breast carcinogenesis, related to the conversion of androgen into oestrogen by aromatase [13]. This enzymatic activity is increased in obese patients due to the expansion of adiposity [48]. However, it is not clear whether testosterone *per se* is directly responsible for promoting breast cancer risk or whether it is just a marker of the dysmetabolism linked to overweight and obesity [49]. This later hypothesis was confirmed in our study by the significant correlation of plasma testosterone with several body and biological markers associated with this dysmetabolism (positively with body weight and ratio of WC/HC, and negatively with HDL-C).

Our trial suffers from several limitations:

1. First, the small numbers of patients divided into different BMI subgroups limited the reliability of the statistical analysis.
2. Second, the determination of biological parameters at one-year follow-up did not permit the characterization of the short-term benefits of our 2-week SPA intervention. Moreover, the one-year time window could explain the weak impact of this intervention on the biological parameters.
3. Third, the mismatches observed between diet consumption and weight changes of patients question the reliability of data collection using the 72-h self-reported diet questionnaire.

Few studies investigating the benefits of physical activity and nutritional interventions in cancer survivors have considered the biological status of the patients in their outcomes. Our data demonstrated that the health changes of patients were mainly related to their body condition and highlighted the importance of evaluating biological and anthropometric status in monitoring cancer survivors.

## 5. Conclusion

To conclude, our study shows that one year after a global multidisciplinary supportive and educational intervention, few anthropometric and biological changes could be attributed to this intervention. It demonstrates that the one-year changes of patients are mainly related to their body mass index (BMI) and confirms the importance of taking into account biological markers of metabolic status in the follow-up of posttherapy breast disease. Among the tools needed for this monitoring, our study highlights the interest of plasma testosterone in the evaluation of recurrence risk. These observations may help reinforce care recommendations for cancer survivors but need to be confirmed on a large population for a more comprehensive approach. Future studies would permit a better understanding of the mechanisms by which such multidisciplinary interventions could interact with breast cancer recurrence and help define the most effective modalities.

## Figures and Tables

**Table 1:** Study population characterization.

Parameter		SPA group (*n* = 57)	CTR group (*n* = 56)	*p* value
		Size or mean ± SD (%) or [mini-max]	Size or mean ± SD (%) or [mini-max]	
Patients' age at allocation		52.0 ± 7.2	51.9 ± 10.6	0.97
		[36–66]	[29–71]	
Menopausal status		Yes = 33 (58%)	Yes = 35 (63%)	0.62
BMI—body mass index (kg/m^2^)		25.4 ± 4.6	25.5 ± 4.4	0.92
		[18.4–35.9]	[18.0–38.7]	
	≤25 kg/m^2^	30 (53%)	27 (48%)	0.37
BMI—class	25–30 kg/m^2^	16 (28%)	22 (39%)	
	>30 kg/m^2^	11 (19%)	7 (13%)	
SF36—global score/100		55 9 ± 15.2	56.8 ± 14.0	0.30
		[19.0–93.0]	[29.0–95.0]	
Surgery for breast cancer		Yes = 57 (100%)	Yes = 55 (98%)	0.50
Radiotherapy		Yes = 54 (95%)	Yes = 54 (96%)	0.98
Hormonotherapy		Yes = 43 (75%)	Yes = 43 (77%)	0.87
Herceptin		Yes = 5 (9%)	Yes = 7 (13%)	0.56
Chemotherapies: number of cycles		6.3 ± 1.1	6.0 ± 0.8	0.29
		[5–15]	[3–9]	

The main covariates of the studied population at allocation are presented with mean ± standard deviation (SD) for Gaussian quantitative variables. Outcomes are shown with 95% confidence intervals. Categorical variables were described using counts by class and frequencies (%). Comparison of outcomes was tested with Student's *t*-test or the Kruskal-Wallis H-test depending on homoscedasticity or normality of distributions. Categorical data were compared with the chi^2^ test. All tests were two-sided, and the nominal level of significance was 5%.

**Table 2:** Diet and Body parameters at allocation.

*Mean* *±* *σ*	*All groups (n* *=* *113)*	BMI (kg/m^2^)			*p* value of BMI effect
		≤25 (*n* = 57)	25–30 (*n* = 38)	>30 (*n* = 18)	
*Diet parameters*					
Total energy intake (TEI) (kcal/d)	*1492* *±* *450*	1540 ± 358	1325 ± 378	1689 ± 678	0.038
Protein intake (g/d)	*63.6* *±* *20.2*	65.3 ± 15.1	58.7 ± 20.0	68.8 ± 30.1	0.86
(% TEI)	*17.3* *±* *4.1*	17.2 ± 3.5	17.9 ± 5.2	16.4 ± 3.1	0.71
Carbohydrate intake (g/d)	*172.6* *±* *61.5*	175.3 ± 54.1	156.8 ± 53.7	197.2 ± 85.1	0.65
(% TEI)	*46.7* *±* *10.4*	45.4 ± 9.5	48.1 ± 12.8	47.8 ± 6.5	0.75
Lipid intake (g/d)	*59.7* *±* *25.4*	63.5 ± 22.3	50.6 ± 23.6	66.8 ± 31.8	0.034
% TEI	*35.5* *±* *8.6*	36.8 ± 8.4	33.5 ± 9.8	35.8 ± 5.0	0.14
*Body parameters*					
Body weight (kg)	*65.2* *±* *12.5*	56.6 ± 6.4	68.5 ± 5.8	85.3 ± 10.7	<10^−7^
Lean mass (LM) (kg)	*42.1* *±* *5.8*	39.6 ± 4.5	43.0 ± 4.8	47.9 ± 6.3	<10^−7^
(%)	*65.2* *±* *6.8*	69.6 ± 5.3	62.9 ± 3.7	56.3 ± 4.5	<10^−7^
Fat mass (FM) (kg)	*23.0* *±* *7.8*	17.2 ± 3.7	25.5 ± 3.1	36.2 ± 5.1	<10^−7^
(%)	*34.6* *±* *6.7*	30.1 ± 5.0	37.3 ± 3.8	43.1 ± 4.4	<10^−7^
Ratio LM/FM	*2.0* *±* *0.6*	2.4 ± 0.6	1.7 ± 0.3	1.3 ± 0.3	<10^−7^
Cell mass (kg)	*25.0* *±* *4.0*	22.8 ± 2.5	25.5 ± 3.3	30.7 ± 3.5	<10^−7^
Total water (l)	*32.9* *±* *3.9*	31.1 ± 2.6	33.2 ± 2.7	38.1 ± 4.6	<10^−7^
(%)	*51.3* *±* *5.4*	55.1 ± 4.0	48.5 ± 3.2	44.9 ± 2.8	<10^−7^
Extracellular water (%)	*24.3* *±* *3.4*	25.7 ± 1.7	23.1 ± 2.1	22.8 ± 6.5	<10^−7^
Intracellular water (%)	*27.1* *±* *2.4*	28.2 ± 1.8	26.1 ± 2.8	25.6 ± 1.2	<10^−7^
Tricipital fold thickness (cm)	*17.4* *±* *8.6*	12.5 ± 5.2	18.8 ± 7.2	29.6 ± 6.4	<10^−7^
Arm circumference (cm)	*30.2* *±* *3.8*	27.7 ± 2.2	31.1 ± 1.7	36.4 ± 3.3	<10^−7^
Waist circumference (WC) (cm)	*84.0* *±* *13.5*	75.4 ± 7.7	86.8 ± 9.0	105.5 ± 8.9	<10^−7^
Hip circumference (HC) (cm)	*101.1* *±* *9.1*	95.0 ± 4.9	103.5 ± 5.5	115.7 ± 5.6	<10^−7^
Ratio WC/HC	*0.83* *±* *0.09*	0.79 ± 0.07	0.84 ± 0.09	0.92 ± 0.08	0.000017

Diet parameters for food intake are expressed in raw value (gram/day) and in % of total energy intake. Body parameters are expressed in raw value (kilogram or liter) and in % of body mass. Comparison of outcomes per BMI group at allocation was tested with one-way analysis of variance (ANOVA). The test was two-sided, and the nominal level of significance was 5%.

**Table 3:** Biological parameters at allocation.

*Mean* *±* *σ*	*All groups* (*n* *=* *113*)	BMI (kg/m^2^)			*p* value of BMI effect
		≤25 (*n* = 57)	25–30 (*n* = 38)	>30 (*n* = 18)	
Glucose (mmol/l)	*5.2* *±* *0.6*	5.1 ± 0. 4	5.2 ± 0.6	5.6 ± 0.8	0.25
HDL-cholesterol (mmol/l)	*2.13* *±* *1.28*	2.35 ± 1.35	1.98 ± 1.25	1.70 ± 0.97	0.0001
Transthyretin (g/l)	*0.26* *±* *0.04*	0.26 ± 0.04	0.26 ± 0.04	0.26 ± 0.04	0.88
C-reactive protein (mg/l)	*2.5* *±* *3. 6*	1.3 ± 1.2	3.2 ± 4.4	5.2 ± 4.9	0.000002
Insulin (mUI/l)	*6.5* *±* *6.2*	4.7 ± 4.4	6.4 ± 4.4	12.1 ± 9.8	0.000013
IGF-1 (*μ*g/l)	*96.4* *±* *49.3*	95.8 ± 45.6	103.5 ± 45.7	84.7 ± 62.6	0.23
Leptin (*μ*g/l)	*5.7* *±* *4.7*	3.5 ± 2.6	6.0 ± 3.0	12.1 ± 6.0	<10^−7^
Adiponectin (mg/l)	*8.1* *±* *5.1*	8.9 ± 5.3	7.6 ± 4.8	6.6 ± 4.4	0.072
Leptin/adiponectin ratio	*1.22* *±* *2.02*	0.53 ± 0.51	1.26 ± 1.28	3.23 ± 3.86	<10^−7^
Testosterone (nmol/l)	*0.82* *±* *0.36*	0.79 ± 0.29	0.83 ± 0.42	0.87 ± 0.38	0.67
CA 15-3 (kU/l)	*18.1* *±* *18.7*	20.1 ± 24.5	14.1 ± 9.0	19.7 ± 8.4	0.014

Plasma biological parameters are expressed in usual unit per liter. Comparison of outcomes per BMI group at allocation was tested with one-way analysis of variance (ANOVA). The test was two-sided, and the nominal level of significance was 5%.

**Table 4:** Variation in diet and body parameters between one-year follow-up and allocation.

		SPA arm (*n* = 55)			CTR arm (*n* = 49)			*p* value effect of		
		≤25 (*n* = 29)	]25–30] (*n* = 15)	>30 (*n* = 11)	≤25 (*n* = 23)	25–30 (*n* = 19)	>30 (*n* = 7)	SPA	Time	BMI
Diet parameters										
Total energy intake	kcal/d	−41.7 ± 400.5	+25.5 ± 556.4	−400.1 ± 527.6	−227.9 ± 362.6	+165.6 ± 410.4	−437.6 ± 955.1	0.91	**0.039**	0.15
		(-0.02%)	(+10.8%)	(−18.7%)	(-12.3%)	(+20.1%)	(−10.3%)			
Protein intake	g/d	−4.6 ± 17.9	+3.2 ± 25.5	−5.8 ± 19.0	−1.78 ± 27.1	+5.1 ± 21.4	−15.4 ± 45.4	0.71	0.24	0.35
		(-3.9%)	(+17.5%)	(-3.1%)	(+6.0%)	(+26.1%)	(−1.4%)			
Carbohydrate intake	g/d	+8.4 ± 52.2	+6.1 ± 63.7	−57.9 ± 78.3	−28.4 ± 54.8	+6.7 ± 51.2	−46.7 ± 121.1	0.84	0.10	0.38
		(+10.1%)	(+16.7%)	(−21.5%)	(−9.8%)	(+7.5%)	(−1.5%)			
Lipid intake	g/d	−5.5 ± 26.4	−1.3 ± 36.4	−12.7 ± 25.2	−9.4 ± 19.8	+13.0 ± 13.0	−24.6 ± 45.4	0.89	0.099	0.15
		(+0.2%)	(+24.0%)	(−13.8%)	(−7.9%)	(+67.3%)	(−21.1%)			

Body parameters										
Body weight	kg	−0.10 ± 2.16	+1.47 ± 4.00	−0.73 ± 5.76	−0.24 ± 2.67	+0.26 ± 3.86	+0.93 ± 2.41	0.56	0.45	**<10^–7^**
		(−0.2%)	(+2.2%)	(−0.3%)	(−0.5%)	(+0.4%)	(+1.1%)			
Lean mass (LM)	%	+1.03 ± 3.63	−0.25 ± 1.66	−0.34 ± 5.85	0.00 ± 3.20	−0.02 ± 3.14	+3.09 ± 7.07	0.18	0.85	**<10^–7^**
		(+1.7%)	(−0.4%)	(−0.0%)	(−0.0%)	(+0.1%)	(+6.0%)			
Fat mass (FM)	%	−0.85 ± 3.50	+0.25 ± 1.66	+0.31 ± 5.90	−0.00 ± 3.20	+0.02 ± 3.14	−1.64 ± 6.05	0.11	0.86	**<10^–7^**
		(−2.7%)	(+0.6%)	(+2.0%)	(0.0%)	(+0.6%)	(-3.6%)			
LM/FM ratio		+0.10 ± 0.39	−0.01 ± 0.14	−0.03 ± 0.37	+0.04 ± 0.40	−0.01 ± 0.29	+0.17 ± 0.39	0.20	0.77	**<10^–7^**
		(+5.0%)	(−0.6%)	(+1.6%)	(+1.7%)	(+0.9%)	(+14.4%)			
Cell mass	kg	+1.34 ± 6.57	+0.95 2.21	+0.54 ± 3.02	−0.83 ± 4.52	−0.66 ± 4.74	+2.15 ± 5.82	0.19	0.34	**<10^–7^**
		(+6.8%)	(+5.2%)	(+2.0%)	(−2.8%)	(−1.7%)	(+7.3%)			
Total water	l	+0.25 ± 1.62	+0.51 ± 1.45	+0.13 ± 2.53	−0.06 ± 1.02	+0.11 ± 1.86	+1.07 ± 5.15	0.22	0.40	**<10^–7^**
		(+0.9%)	(+1.7%)	(+0.8%)	(−0.1%)	(+0.5%)	(+3.2%)			
Extracellular water	%	+0.51 ± 1.72	−0.67 ± 1.67	+0.02 ± 2.33	+1.14 ± 4.1	0.98 ± 3.72	−3.97 ± 10.72	0.80	0.86	**<10^–7^**
		(+2.0%)	(−2.3%)	(+0.6%)	(+4.9%)	(+4.3%)	(−7.2%)			
Intracellular water	%	+2.21 ± 9.32	+0.61 ± 1.32	−0.08 ± 1.99	−0.35 ± 4.76	−0.59 ± 4.68	+1.65 ± 4.55	0.28	0.53	**0.00012**
		(+7.7%)	(+3.2%)	(−0.2%)	(−1.3%)	(−1.9%)	(+7.0%)			
Tricipital fold thickness	cm	+0.46 ± 3.74	−0.57 ± 7.78	−3.25 ± 5.41	+0.23 ± 5.05	+2.98 ± 5.60	−2.51 ± 4.95	0.36	0.69	**<10^–7^**
		(+10.7%)	(+1.5%)	(−7.5%)	(+14.1%)	(+25.3%)	(−6.3%)			
Arm circumference	cm	−0.10 ± 1.77	−1.00 ± 1.49	−1.46 ± 1.94	−0.46 ± 1.71	−0.32 ± 2.16	−0.93 ± 2.01	0.58	0.32	**<10^–7^**
		(−0.3%)	(−3.1%)	(−3.6%)	(−1.7%)	(−1.1%)	(−2.4%)			
Waist circumference (WC)	cm	−2.93 ± 4.41	+0.43 ± 3.68	−1.09 ± 5.00	−0.46 ± 5.65	+1.45 ± 8.51	−3.36 ± 6.24	0.73	0.81	**<10^–7^**
		(−3.7%)	(−0.4%)	(−1.0%)	(−0.4%)	(+2.7%)	(−3.0%)			
Hip circumference (HC)	cm	−0.59 ± 2.80	1.77 ± 2.83	0.23 ± 5.85	−1.30 ± 3.56	−0.13 ± 3.71	2.79 ± 5.53	0.80	0.66	**<10^–7^**
		(−0.6%)	(+1.7%)	(+0.4%)	(−1.1%)	(−0.1%)	(2.5%)			
WC/HC ratio		−0.03 ± 00.05	−0.01 ± 0.02	−0.01 ± 0.07	+0.01 ± 0.05	+0.2 ± 0.08	−0.05 ± 0.06	0.66	0.61	**<10^–7^**
		(−2.9%)	(−1.5%)	(−1.1%)	(+0.9%)	(+3.1%)	(−4.9%)			

Variation for each parameter is expressed in raw value (one-year follow-up value *minus* allocation value) and in percentage of the allocation value: + sign indicates an increase and –sign indicates a decrease. Two-way ANOVA was used to compare longitudinal variations between allocation arms (SPA effect), or one-year follow-up (time effect), or BMI groups (BMI effect), but without interaction test because of unequal class sizes. All tests were two-sided, and the nominal level of significance was 5%. Significant *p* values are indicated in bold.

**Table 5:** Variation in biological parameters between one-year follow-up and allocation.

		SPA arm (*n* = 55)			CTR arm (*n* = 49)			*p* value effect of		
		≤25	25–30	>30	≤25	25–30	>30	SPA	Time	BMI
		(*n* = 29)	(*n* = 15)	(*n* = 11)	(*n* = 23)	(*n* = 19)	(*n* = 7)			
Glucose	mmol/l	0.007 ± 0.446	0.459 ± 0.748	0.749 ± 1.358	−0.089 ± 0.326	0.226 ± 0.394	1.75 ± 2.775	0.23	**0.04**	**<10^–6^**
		(0.6%)	(8.9%)	(15.9%)	(−1.6%)	(4.4%)	(25.9%)			
HDL-cholesterol	mmol/l	−0.438 ± 1.471	−0.241 ± 1.34	−0.542 ± 1.31	−0.436 ± 1.384	−0.333 ± 1.635	−0.028 ± 0.198	0.41	**0.027**	**0.007**
		(−5.6%)	(3.4%)	(−13%)	(−4.8%)	(7%)	(−1.3%)			
Transthyretin	g/l	0.001 ± 0.038	0.009 ± 0.026	−0.005 ± 0.027	0.001 ± 0.033	−0.007 ± 0.031	0.002 ± 0.051	**0.041**	0.75	0.79
		(1%)	(3.5%)	(−1.5%)	(0.8%)	(−2%)	(3.2%)			
C-reactive protein	mg/l	−0.146 ± 1.142	0.238 ± 2.818	−0.127 ± 1.481	0.264 ± 1.168	−0.135 ± 4.736	−1 ± 5.545	0.11	0.73	**<10^–7^**
		(11.1%)	(27.3%)	(8.9%)	(41.8%)	(41.1%)	(−6.6%)			
Insulin	mUI/l	0.17 ± 3.79	1.97 ± 4.59	2.51 ± 10.19	0.22 ± 6.58	4.47 ± 10.51	4.53 ± 6.24	0.41	**0.035**	**<10^–7^**
		(25.7%)	(115.7%)	(58.4%)	(36.1%)	(50.9%)	(78.4%)			
IGF-1	*μ*g/l	−0.79 ± 27.51	−26.61 ± 27.25	−18.78 ± 23.9	−13.18 ± 39.9	−12.41 ± 34.83	9.13 ± 16.55	0.32	0.072	0.31
		(6.3%)	(−19.7%)	(−24%)	(−4.2%)	(−12.2%)	(16.5%)			
Leptin	*μ*g/l	−0.03 ± 1.57	−0.02 ± 2.72	−2.42 ± 6.24	0.12 ± 2.38	1.64 ± 6.28	−0.93 ± 3.78	0.66	0.81	**<10^–7^**
		(15.6%)	(9%)	(−2.4%)	(21.7%)	(23.6%)	(−7.4%)			
Adiponectin	mg/l	2.33 ± 4.19	1.29 ± 2.3	0.57 ± 1.3	0.99 ± 2.51	1.15 ± 2.65	0.65 ± 1.01	0.33	0.082	**0.022**
		(32.6%)	(17.3%)	(18%)	(13.9%)	(11.5%)	(5.6%)			
Leptin/adiponectin ratio		−0.1 ± 0.27	−0.08 ± 0.52	−1.68 ± 2.92	0.13 ± 1.21	1.2 ± 4.07	−0.27 ± 0.29	0.73	0.91	**2 × 10^–6^**
		(−2.9%)	(−0.4%)	(−18%)	(24.2%)	(16.6%)	(−13.6%)			
Testosterone	nmol/l	−0.033 ± 0.299	−0.013 ± 0.098	0.045 ± 0.347	−0.051 ± 0.244	0.015 ± 0.332	0.029 ± 0.757	0.086	0.83	0.27
		(−3.6%)	(5%)	(10.7%)	(−5%)	(3.4%)	(21.8%)			
CA 15-3	kU/l	2.32 ± 3.08	0.62 ± 1.78	0.27 ± 1.71	−5.32 ± 28.45	1.56 ± 2.06	2.71 ± 1.48	**0.04**	0.68	**0.07**
		(18.2%)	(6.8%)	(4.7%)	(1.4%)	(12.5%)	(14%)			

Variation for each parameter is expressed in raw value (one-year follow-up value *minus* allocation value) and in percentage of the allocation value: + sign indicates an increase and – sign indicates a decrease. Two-way ANOVA was used to compare longitudinal variations between allocation arms (SPA effect), or one-year follow-up (time effect), or BMI groups (BMI effect), but without interaction test because of unequal class sizes. All tests were two-sided, and the nominal level of significance was 5%. Significant *p* values are indicated in bold.

**Table 6:** Prognostic value of biological parameters on disease-free survival over 7 years.

Parameters at allocation (*n* = 111)	Median (quartiles)	Threshold		
		≤1^st^ quartile	≤ Median	≤3^rd^ quartile
Cholesterol-HDL (mmol/l)	1.78 [1.46–2.13]	*p*=0.64	*p*=0.22	*p*=0.047^(+)^
Testosterone (nmol/l)	0.7 [0.7–0.9]	ND	*p*=0.049^(−)^	*p*=0.001^(−)^
CA 15-3 (kU/l)	14 [10–20]	*p*=0.28	*p*=0.07^(−)^	*p*=0.03^(−)^

Association of biological parameters at allocation with the recurrence risk was tested using a two-sided chi^2^ test. The nominal level of significance was 5%. + sign indicates that high values are in favour of a better prognosis, while – sign indicates that these high values worsen prognosis.

## Data Availability

The data used to support the findings of this study are available from the corresponding author upon request.

## Additional Points

*Highlights*. (1) After breast cancer treatment completion, changes in anthropometric and biological parameters are mainly dependent on the patient's BMI level. (2) A reinforcement of insulin resistance is observed in overweight and obese patients after one-year treatment completion, independently of physical activity and nutritional intervention. (3) Testosterone plasma levels at the time of treatment completion are associated with recurrence risk in patients receiving adjuvant hormonotherapy.

## Supplementary Materials

Supplementary Materials Table 1: diet, body, and biological parameters for the SPA arm at allocation and one-year follow-up. Table 2: diet, body, and biological parameters for the CTR arm at allocation and one-year follow-up. Overall protocol design.
