# Supplementary material for: Effectiveness of a Global Multidisciplinary Supportive and Educational Intervention in Thermal Resort on Anthropometric and Biological Parameters, and the Disease-Free Survival after Breast Cancer Treatment Completion (PACThe)
Source: J Oncol. 2020 May 5;2020:4181850. doi: 10.1155/2020/4181850 (PMC7222596; doi:10.1155/2020/4181850)
Supplement: Supplementary Materials — Table 1: diet, body, and biological parameters for the SPA arm at allocation and one-year follow-up. Table 2: diet, body, and biological parameters for the CTR arm at allocation and one-year follow-up. Overall protocol design. [file 4181850.f1.pdf]

Table 1sup: Parameters for SPA arm at allocation and one-year follow-up

		Mean ± σ (n=55) [mini-max]	BMI (kg/m²) at allocation			*Mean ± σ (n=55) [mini-max]*	BMI (kg/m²) at one-year follow-up		
			≤ 25 (n=29)	]25 - 30] (n=15)	> 30 (n=11)		≤ 25 (n=29)	]25 - 30] (n=15)	> 30 (n=11)
**Diet parameters**									
Total Energy Intake (TEI)	(kcal/d)	*1489 ± 374 [695-2724]*	1477 ± 254 [1071-2017]	1381 ± 430 [695-2028]	1681 ± 475 [972-2724]	*1383 ± 360 [821-2496]*	1433 ± 303 [955-2068]	1363 ± 435 [834-2496]	1280 ± 360 [821-1840]
Protein intake	(g/d)	*64.4 ± 18.2 [26-123]*	65.4 ± 14.1 [41-91]	59.8 ± 19.3 [26-90]	68.1 ± 24.0 [41-123]	*60.8 ± 16.5 [22-108]*	60.1 ± 15.6 [34-91]	61.0 ± 17.6 [30-108]	62.3 ± 17.3 [22-91]
	% TEI	*17.5 ± 4.0 [8-33]*	17.8 ± 3.2 [10-26]	17.9 ± 5.3 [11-33]	16.2 ± 3.2 [8-19]	*18.5 ± 5.8 [10-50]*	16.9 ± 3.5 [10-28]	20.6 ± 8.4 [14-50]	20.0 ± 5.2 [10-30]
Carbohydrate intake	(g/d)	*169.6 ± 54.7 [71-362]*	161.4 ± 35.8 [92-267]	162.8 ± 57.7 [71-264]	201.6 ± 77.3 [100-362]	*162.2 ± 46.6 [70-310]*	169.2 ± 41.3 [88-306]	162.1 ± 50.8 [107-310]	143.7 ± 48.5 [70-216]
	% TEI	*46.1 ± 9.1 [28-66]*	44.0 ± 8.0 [28-60]	47.6 ± 11.3 [29-64]	49.9 ± 6.5 [42-66]	*46.9 ± 7.6 [30-62]*	47.1 ± 7.2 [30-62]	48.3 ± 7.9 [31-62]	44.6 ± 7.7 [34-61]
Lipid intake	(g/d)	*59.9 ± 22.6 [21-114]*	62.1 ± 18.8 [30-114]	53.8 ± 28.8 [21-111]	62.7 ± 19.6 [36-114]	*54.3 ± 19.8 [16-106]*	57.0 ± 17.4 [33-96]	52.1 ± 24.1 [16-106]	50.0 ± 17.8 [30-95]
	% TEI	*35.8 ± 8.4 [14-55]*	37.3 ± 7.8 [22-52]	34.4 ± 10.9 [14-55]	34.0 ± 4.1 [26-39]	*34.9 ± 6.7 [14-53]*	35.7 ± 6.4 [23-53]	33.5 ± 7.7 [14-49]	35.0 ± 5.4 [27-46]
**Body parameters**									
Body weight	(kg)	*65.7 ± 14.2 [47-115]*	55.9 ± 5.7 [47-73]	69.6 ± 6.0 [61-78.5]	86.9 ± 12.7 [70-115]	*65.9 ± 14.0 [47.5-112]*	55.9 ± 6.1 [47.5-74.5]	70.5 ± 6.8 [61.5-83.5]	86.2 ± 10.5 [69-112]
Lean Mass (LM)	(kg)	*42.6 ± 6.2 [32-63.5]*	39.3 ± 3.9 [32-49.5]	43.9 ± 4.6 [37.4-53.5]	49.5 ± 7.2 [37.5-63.5]	*42.8 ± 5.8 [32.8-60.9]*	39.6 ± 3.8 [32.8-48.4]	43.8 ± 3.8 [38.1-50.4]	49.7 ± 5.9 [37-60.9]
	(%)	*65.5 ± 6.6 [52.7-81.8]*	69.6 ± 5.3 [56.4-81.8]	63.0 ± 3.7 [58.3-71.1]	58.0 ± 4.3 [52.7-66.8]	*65.9 ± 7.2 [53.6-81.9]*	71.0 ± 5.1 [61.3-81.9]	62.4 ± 4.1 [57.8-71]	57.7 ± 3.5 [53.6-65.1]
Fat Mass (FM)	(kg)	*23.0 ± 8.2 [9.1-46.2]*	16.9 ± 3.4 [9.1-23.5]	25.8 ± 3.3 [17.9-30]	35.6 ± 5.2 [30.1-46.2]	*23.3 ± 9.2 [8.8-50.6]*	16.4 ± 4.1 [8.8-26.1]	26.7 ± 4.6 [18.1-35.2]	36.5 ± 6.0 [27-50.6]
	(%)	*34.2 ± 6.5 [18.2-47.3]*	29.9 ± 4.6 [18.2-40.2]	37.0 ± 3.7 [28.9-41.7]	42.0 ± 4.3 [33.2-47.3]	*34.1 ± 7.2 [18.1-46.4]*	28.9 ± 5.1 [18.1-38.7]	37.6 ± 4.1 [29-42.2]	42.3 ± 3.5 [34.6-46.4]
Ratio LM/FM		*2.0 ± 0.6 [1.1-4.5]*	2.4 ± 0.6 [1.5-4.5]	1.7 ± 0.3 [1.4-2.5]	1.4 ± 0.3 [1.1-2.0]	*2.1 ± 0.8 [1.2-4.5]*	2.6 ± 0.7 [1.6-4.5]	1.7 ± 0.3 [1.4-2.5]	1.4 ± 0.2 [1.2-1.9]
Cell Mass	(kg)	*25.1 ± 4.3 [15-36.8]*	22.8 ± 2.0 [19.7-27.7]	25.5 ± 4.3 [15-36.7]	30.8 ± 3.7 [24.4-36.8]	*26.0 ± 5.7 [16.1-53.1]*	24.2 ± 6.2 [16.1-53.1]	25.7 ± 2.3 [22-29.1]	31.3 ± 4.3 [23.9-39.4]
Total water	(l)	*33.2 ± 4.1 [27.4-48.2]*	31.1 ± 2.3 [27.4-35.9]	33.6 ± 3.2 [28.8-41.4]	38.3 ± 4.7 [30.5-48.2]	*33.5 ± 3.8 [27.1-46.5]*	31.5 ± 2.4 [27.1-36.8]	33.6 ± 2.5 [30-37.5]	38.4 ± 3.8 [30.4-46.5]
	(%)	*51.5 ± 5.7 [40.2-63.6]*	55.6 ± 4.0 [49.2-63.6]	48.3 ± 3.3 [44.1-56.1]	45.1 ± 2.8 [40.2-50.7]	*51.7 ± 6.5 [41.7-66.9]*	56.5 ± 4.7 [47.2-66.9]	47.7 ± 3.4 [44-56.2]	44.7 ± 2.1 [41.7-49.5]
Extracellular water	(%)	*24.3 ± 2.7 [19.4-32.6]*	25.9 ± 1.6 [23.2-29.1]	23.5 ± 2.8 [21.1-32.6]	21.3 ± 1.2 [19.4-23.7]	*24.4 ± 3.0 [19.8-33.1]*	26.3 ± 2.3 [22.4-33.1]	22.9 ± 1.9 [20.9-27.2]	21.3 ± 1.6 [19.8-26]
Intracellular water	(%)	*27.0 ± 2.7 [15-32.7]*	28.5 ± 1.6 [25.4-32.6]	25.6 ± 3.6 [15-32.7]	25.3 ± 0.9 [23.5-27.1]	*28.1 ± 7.5 [18.4-78.2]*	30.6 ± 9.6 [22.1-78.2]	25.7 ± 2.3 [18.4-29.6]	25.2 ± 1.8 [23-30.1]

	Mean ± σ (n=55) [mini-max]	BMI (kg/m²) at allocation			*Mean ± σ (n=55) [mini-max]*	BMI (kg/m²) at one-year follow-up		
		≤ 25 (n=29)	]25 - 30] (n=15)	> 30 (n=11)		≤ 25 (n=29)	]25 - 30] (n=15)	> 30 (n=11)
Tricipital fold thickness (cm)	*18.2 ± 9.8 [4.3-43]*	12.3 ± 5.4 [4.3-24]	20.1 ± 8.5 [8.3-43]	30.8 ± 6.6 [13.6-38.7]	*17.6 ± 8.1 [4-37]*	13.0 ± 5.7 [4-27.3]	19.4 ± 6.2 [8-28]	27.6 ± 5.1 [18-37]
Arm Circumference (cm)	*30.4 ± 4.3 [24-42.5]*	27.5 ± 2.4 [24-34.5]	31.6 ± 1.7 [28-34.5]	36.6 ± 3.7 [30-42.5]	*29.9 ± 3.8 [24-39.5]*	27.5 ± 2.3 [24-33]	30.8 ± 1.9 [27.5-34]	35.1 ± 2.8 [30-39.5]
Waist Circumference (WC) (cm)	*84.8 ± 14.0 [63-121]*	75.0 ± 7.3 [63-89]	89.4 ± 8.7 [76.5-108]	104.8 ± 7.7 [95-121]	*83.4 ± 15.3 [63.5-123]*	72.1 ± 7.1 [63.5-87.5]	90.1 ± 10.8 [74.5-116]	103.7 ± 8.2 [88-123]
Hip Circumference (HC) (cm)	*101.1 ± 9.8 [85-125]*	94.4 ± 5.3 [85-105]	103.5 ± 5.2 [92.5-112]	116.1 ± 5.1 [110-125]	*101.3 ± 10.1 [86-122]*	93.8 ± 5.2 [86-107]	104.7 ± 5.4 [96-114]	116.4 ± 3.3 [110-122]
Ratio WC/HC	*0.84 ± 0.09 [0.67-1.07]*	0.79 ± 0.07 [0.67-0.97]	0.87 ± 0.08 [0.76-1.00]	0.91 ± 0.07 [0.80-1.07]	*0.82 ± 0.09 [0.69-1.01]*	0.77 ± 0.06 [0.69-0.93]	0.86 ± 0.08 [0.74-1.01]	0.90 ± 0.07 [0.77-1.00]
**Biological parameters**								
Glucose (mmol/l)	*5.1 ± 0.6 [3.9-7.1]*	5.1 ± 0.4 [3.9-5.8]	5.1 ± 0.5 [4.5-6.2]	5.5 ± 0.8 [4.61-7.1]	*5.4 ± 0.9 [4.3-8.4]*	5.1 ± 0.4 [4.3-5.8]	5.5 ± 1.0 [4.5-8.4]	6.1 ± 1.2 [4.5-8.2]
HDL Cholesterol (mmol/l)	*2.23 ± 1.31 [1.16-6.79]*	2.54 ± 1.39 [1.29-6.79]	1.97 ± 1.08 [1.28-5.96]	1.82 ± 1.21 [1.16-5.6]	*1.83 ± 0.50 [0.94-3.56]*	2.05 ± 0.52 [0.94-3.56]	1.73 ± 0.33 [1.01-2.39]	1.35 ± 0.09 [1.16-1.51]
Transthyretin (g/l)	*0.25 ± 0.03 [0.20-0.38]*	0.25 ± 0.04 [0.20-0.38]	0.25 ± 0.03 [0.22-0.29]	0.25 ± 0.03 [0.21-0.31]	*0.25 ± 0.04 [0.19-0.35]*	0.25 ± 0.04 [0.19-0.35]	0.26 ± 0.04 [0.19-0.31]	0.24 ± 0.03 [0.19-0.29]
C-reactive protein (mg/l)	*2.1 ± 2.3 [0.5-13.9]*	1.3 ± 1.1 [0.5-5.7]	2.5 ± 1.9 [0.5-8.3]	3.9 ± 3.5 [0.8-13.9]	*2.1 ± 2.5 [0.6-12.3]*	1.1 ± 0.7 [0.6-3.7]	3.0 ± 2.8 [0.6-8.7]	3.8 ± 3.4 [0.8-12.3]
Insuline (mUI/l)	*6.4 ± 7.1 [1.5-47.5]*	4.2 ± 3.8 [1.5-22.2]	5.9 ± 4.6 [1.5-19.1]	12.8 ± 11.7 [4.7-47.5]	*7.7 ± 7.1 [1.5-34.5]*	4.5 ± 3.1 [1.5-11.1]	8.2 ± 4.0 [1.5-14.4]	15.3 ± 10.5 [2-34.5]
IGF-1 (µg/l)	*94.4 ± 51.8 [8.7-284]*	85.1 ± 40.4 [28.3-215]	118.5 ± 43.0 [57.9-198]	85.3 ± 73.6 [8.7-284]	*81.1 ± 40.3 [1.8-200]*	81.4 ± 33.2 [33.5-177]	91.9 ± 35.4 [40.9-153]	66.5 ± 55.0 [1.8-200]
Leptin (µg/l)	*5.8 ± 5.2 [0.7-32]*	3.1 ± 2.2 [0.7-9]	6.3 ± 2.5 [2.5-11.3]	12.3 ± 7.2 [5-32]	*5.5 ± 3.9 [0.6-16.6]*	3.0 ± 2.2 [0.6-10.5]	6.9 ± 2.9 [2.2-13.8]	9.9 ± 3.8 [5.4-16.6]
Adiponectin (mg/l)	*8.3 ± 5.4 [1-36]*	9.8 ± 6.1 [4.5-36]	7.7 ± 4.2 [1.66-20.2]	5.3 ± 3.4 [1-13.4]	*9.9 ± 6.3 [1.06-37.6]*	12.1 ± 7.0 [4.05-37.6]	9.0 ± 4.7 [1.7-18.4]	5.8 ± 3.1 [1.06-12.5]
Ratio leptin /adiponectin	*1.36 ± 2.60 [0.07-17.5]*	0.40 ± 0.40 [0.07-1.67]	1.16 ± 1.00 [0.25-4.34]	4.08 ± 4.65 [0.63-17.5]	*0.99 ± 1.30 [0.04-7.45]*	0.31 ± 0.23 [0.04-0.99]	1.20 ± 1.04 [0.17-4.24]	2.40 ± 1.81 [0.46-7.45]
Testosterone (nmol/l)	*0.77 ± 0.28 [0.14-1.6]*	0.78 ± 0.28 [0.18-1.6]	0.73 ± 0.21 [0.14-1.2]	0.81 ± 0.35 [0.29-1.5]	*0.77 ± 0.30 [0.02-2.1]*	0.78 ± 0.33 [0.09-2.1]	0.68 ± 0.12 [0.27-0.8]	0.86 ± 0.34 [0.02-1.4]
CA 15-3 (kU/l)	*15.2 ± 8.4 [5-54]*	14.6 ± 6.2 [6-34]	14.5 ± 10.8 [7-54]	17.8 ± 8.9 [5-37]	*16.2 ± 7.1 [6-36]*	17.1 ± 7.3 [6-36]	12.8 ± 3.8 [9-22]	18.1 ± 8.1 [6-35]

Diet parameters for food intake are expressed in raw value (gram / day) and in % of total energy intake. Body parameters are expressed in raw value (kilogram or liter) and in % of body mass. Plasma biological parameters are expressed in usual unit per liter.

Table 2Sup: Parameters for CTR arm at allocation and one-year follow-up

		Mean ± σ (n=49) [mini-max]	BMI (kg/m^2) at allocation			*Mean ± σ (n=49) [mini-max]*	BMI (kg/m^2) at one-year follow-up		
			≤ 25 (n=23)	]25 - 30] (n=19)	> 30 (n=7)		≤ 25 (n=23)	]25 - 30] (n=19)	> 30 (n=7)
**Diet parameters**									
Total Energy Intake (TEI)	(kcal/d)	*1494 ± 516<br>[469-3623]*	1611 ± 436<br>[762-2575]	1284 ± 329<br>[738-1948]	1701 ± 909<br>[469-3623]	*1365 ± 398<br>[196-2369]*	1354 ± 415<br>[196-2369]	1415 ± 341<br>[903-2076]	1263 ± 457<br>[611-2072]
Protein intake	(g/d)	*62.9 ± 22<br>[17-154]*	65.1 ± 16.1<br>[28-96]	57.9 ± 20.4<br>[17-95]	69.9 ± 37.7<br>[21-154]	*60.3 ± 17.8<br>[30-125]*	62.4 ± 19.6<br>[35-125]	60.1 ± 13.9<br>[30-94]	54.4 ± 19.5<br>[32-94]
	% TEI	*17.1 ± 4.3<br>[9-32]*	16.5 ± 3.6<br>[13-26]	18.0 ± 5.1<br>[9-32]	16.9 ± 2.8<br>[11-20]	*18.0 ± 5.4<br>[11-39]*	18.4 ± 6.4<br>[11-39]	17.4 ± 4.5<br>[13-31]	18.4 ± 3.7<br>[12-22]
Carbohydrate intake	(g/d)	*175.6 ± 67.5<br>[26-371]*	190.7 ± 65.5<br>[51-347]	152.4 ± 50.2<br>[26-246]	190.1 ± 95.6<br>[44-371]	*157.7 ± 54.5<br>[48-296]*	159.5 ± 50.9<br>[50-296]	160.8 ± 50.7<br>[48-243]	143.4 ± 71.3<br>[73-259]
	% TEI	*47.3 ± 11.6<br>[8-75]*	47 ± 10.7<br>[22-67]	48.6 ± 13.8<br>[8-75]	44.4 ± 4.9<br>[38-53]	*45.9 ± 9.1<br>[19-61]*	46.1 ± 8.6<br>[20-59]	45.6 ± 9.7<br>[19-61]	45.7 ± 8.7<br>[31-59]
Lipid intake	(g/d)	*59.5 ± 27.9<br>[13-169]*	65.0 ± 25.6<br>[31-125]	48.3 ± 18.6<br>[13-75]	73.3 ± 44.0<br>[23-169]	*55.0 ± 19.4<br>[16-103]*	54.0 ± 18.9<br>[33-103]	58.5 ± 19.5<br>[27-97]	48.7 ± 18.8<br>[16-82]
	% TEI	*35.3 ± 8.8<br>[15-53]*	36.3 ± 9.0<br>[19-52]	32.9 ± 8.9<br>[15-53]	38.6 ± 5.0<br>[28-44]	*35.5 ± 6.9<br>[24-50]*	34.4 ± 6.0<br>[24-47]	36.6 ± 7.5<br>[25-50]	35.9 ± 7.3<br>[24-47]
**Body parameters**									
Body weight	(kg)	*64.6 ± 10.4<br>[49-89.5]*	57.4 ± 7.0<br>[49-75.3]	67.7 ± 5.5<br>[57.3-78]	82.7 ± 5.3<br>[72-89.5]	*65.9 ± 11.2<br>[43.6-90.5]*	58.1 ± 7.9<br>[43.6-77]	68.8 ± 6.1<br>[59.6-80]	83.6 ± 5.8<br>[73.5-90.5]
Lean Mass (LM)	(kg)	*41.6 ± 5.2<br>[29.6-52.8]*	39.9 ± 5.2<br>[29.6-52.8]	42.4 ± 4.8<br>[34.4-51]	45.5 ± 3.4<br>[42.3-50.9]	*42.1 ± 5.8<br>[27.5-61]*	39.6 ± 5.3<br>[27.5-51.3]	43.0 ± 4.2<br>[33.9-49.9]	47.4 ± 6.8<br>[41.9-61]
	(%)	*64.9 ± 7.0<br>[49.2-79.7]*	69.6 ± 5.4<br>[59.8-79.7]	62.8 ± 3.8<br>[56.4-72.8]	53.7 ± 3.5<br>[49.2-58.9]	*64.6 ± 6.8<br>[47.2-79.8]*	68.6 ± 5.9<br>[58.7-79.8]	62.7 ± 3.8<br>[54.7-69.8]	56.8 ± 6.9<br>[47.2-67.8]
Fat Mass (FM)	(kg)	*23.1 ± 7.5<br>[11.7-43.7]*	17.5 ± 4.0<br>[11.7-25.1]	25.3 ± 2.9<br>[18-30.7]	37.2 ± 4.8<br>[29.6-43.7]	*23.7 ± 7.7<br>[10.3-47.8]*	18.3 ± 4.7<br>[10.3-26.4]	25.6 ± 3.8<br>[18.2-33.4]	36.2 ± 6.6<br>[29-47.8]
	(%)	*35.0 ± 6.8<br>[20.3-50.8]*	30.4 ± 5.4<br>[20.3-40.2]	37.5 ± 3.8<br>[27.2-43.6]	44.9 ± 3.9<br>[39.4-50.8]	*35.4 ± 6.8<br>[20.2-52.8]*	31.4 ± 5.9<br>[20.2-41.3]	37.3 ± 3.8<br>[30.2-45.3]	43.2 ± 6.9<br>[32-52.8]
Ratio LM/FM		*1.98 ± 0.66<br>[0.97-3.93]*	2.40 ± 0.64<br>[1.49-3.93]	1.71 ± 0.31<br>[1.29-2.68]	1.21 ± 0.16<br>[0.97-1.43]	*1.95 ± 0.65<br>[0.89-3.95]*	2.32 ± 0.71<br>[1.42-3.95]	1.71 ± 0.28<br>[1.21-2.31]	1.38 ± 0.40<br>[0.89-2.12]
Cell Mass	(kg)	*24.8 ± 3.6<br>[16.4-36.3]*	22.9 ± 2.9<br>[16.4-28.6]	25.5 ± 2.4<br>[22-31.9]	30.6 ± 3.1<br>[26.5-36.3]	*24.9 ± 5.5<br>[6.9-44.9]*	22.3 ± 4.2<br>[6.9-28.4]	25.1 ± 4.1<br>[9.6-29.2]	32.4 ± 5.7<br>[26.6-44.9]
Total water	(l)	*32.6 ± 3.6<br>[24.3-47]*	31.1 ± 2.9<br>[24.3-37.5]	32.8 ± 2.2<br>[29.4-36.8]	37.7 ± 4.3<br>[33.9-47]	*33.0 ± 4.0<br>[24.1-50.8]*	31.2 ± 2.8<br>[24.1-36.1]	33.2 ± 1.9<br>[29.2-36.5]	38.7 ± 5.8<br>[33.3-50.8]
	(%)	*51.0 ± 5.0<br>[40.5-61.6]*	54.5 ± 3.9<br>[48.8-61.6]	48.7 ± 3.2<br>[42.4-55.3]	44.5 ± 2.8<br>[40.5-48.9]	*50.9 ± 5.4<br>[39.2-63]*	54.1 ± 4.2<br>[46.9-63]	48.6 ± 3.8<br>[40.9-56.5]	46.3 ± 5.7<br>[39.2-56.4]
Extracellular water	(%)	*24.4 ± 4.0<br>[20-49.1]*	25.5 ± 1.9<br>[22.6-30.4]	22.8 ± 1.2<br>[20.2-24.9]	25.2 ± 9.8<br>[20-49.1]	*24.5 ± 4.1<br>[19-42.5]*	26.1 ± 3.9<br>[22.2-42.5]	23.6 ± 4.0<br>[19.6-39.4]	21.3 ± 1.7<br>[19-23.7]
Intracellular water	(%)	*27.1 ± 2.0<br>[23.5-33.8]*	27.9 ± 1.9<br>[23.5-32.6]	26.4 ± 1.9<br>[23.7-33.8]	26.1 ± 1.6<br>[24.6-29.2]	*26.7 ± 4.7<br>[6.5-37.3]*	27.4 ± 5.0<br>[6.5-37.3]	25.8 ± 4.4<br>[9.2-32.2]	27.1 ± 3.8<br>[23.1-35]

	Mean ± σ (n=49) [mini-max]	BMI (kg/m²) at allocation			*Mean ± σ (n=49) [mini-max]*	BMI (kg/m²) at one-year follow-up		
		≤ 25 (n=23)	]25 - 30] (n=19)	> 30 (n=7)		≤ 25 (n=23)	]25 - 30] (n=19)	> 30 (n=7)
Tricipital fold thickness (cm)	*16.6 ± 7.2 [4.7-32.6]*	12.7 ± 4.9 [4.7-24]	17.8 ± 5.9 [6-30]	27.6 ± 5.5 [17-32.6]	*17.8 ± 6.3 [7.8-32]*	13.2 ± 3.5 [7.8-20.6]	20.7 ± 5.3 [8-32]	25.1 ± 3.7 [20.7-32]
Arm Circumference (cm)	*30.0 ± 3.2 [24.5-40]*	27.9 ± 1.9 [24.5-31.5]	30.7 ± 1.6 [27-33]	36.1 ± 2.3 [32.5-40]	*29.9 ± 3.7 [22.5-38]*	27.7 ± 2.7 [22.5-33]	30.6 ± 2.9 [24.5-38]	35.1 ± 1.9 [32-37]
Waist Circumference (WC) (cm)	*83.2 ± 13.0 [60-121]*	75.9 ± 8.1 [62-89]	84.8 ± 8.7 [60-101]	106.5 ± 10.3 [91.5-121]	*84.2 ± 12.1 [64-120]*	76.8 ± 8.3 [64-93]	86.2 ± 7.2 [75-99]	103.1 ± 10.2 [87-120]
Hip Circumference (HC) (cm)	*101.1 ± 8.2 [88-125]*	95.6 ± 4.4 [88-104]	103.5 ± 5.7 [93.5-117]	114.9 ± 6.1 [104-125]	*101.7 ± 10.0 [85-132]*	95.0 ± 6.0 [85-105]	103.9 ± 5.5 [95.5-115]	117.7 ± 9.2 [105-132]
Ratio WC/HC	*0.82 ± 0.09 [0.61-1.04]*	0.79 ± 0.07 [0.69-0.97]	0.82 ± 0.09 [0.61-1]	0.93 ± 0.08 [0.76-1.04]	*0.83 ± 0.08 [0.68-0.97]*	0.81 ± 0.07 [0.71-0.97]	0.83 ± 0.07 [0.68-0.95]	0.88 ± 0.08 [0.73-0.96]
**Biological parameters**								
Glucose (mmol/l)	*5.3 ± 0.5 [4.2-7.2]*	5.2 ± 0.4 [4.39-5.9]	5.3 ± 0.6 [4.2-6.5]	5.7 ± 0.8 [4.9-7.2]	*5.6 ± 1.6 [4.17-15]*	5.0 ± 0.4 [4.17-5.73]	5.5 ± 0.6 [4.4-7.06]	7.3 ± 3.3 [4.6-15]
HDL Cholesterol (mmol/l)	*2.02 ± 1.24 [0.85-6.81]*	2.16 ± 1.27 [0.85-6.81]	2.01 ± 1.37 [0.92-6.35]	1.53 ± 0.25 [1.25-1.87]	*1.80 ± 0.52 [0.88-3.54]*	1.88 ± 0.39 [0.88-2.43]	1.79 ± 0.66 [1.04-3.54]	1.54 ± 0.28 [1.25-1.93]
Transthyretin (g/l)	*0.27 ± 0.04 [0.18-0.37]*	0.26 ± 0.04 [0.19-0.34]	0.27 ± 0.05 [0.18-0.37]	0.27 ± 0.05 [0.21-0.33]	*0.26 ± 0.04 [0.19-0.37]*	0.26 ± 0.05 [0.2-0.37]	0.26 ± 0.04 [0.19-0.33]	0.27 ± 0.04 [0.2-0.32]
C-reactive protein (mg/l)	*3.0 ± 4.5 [0-21.2]*	1.4 ± 1.3 [0.5-6.6]	3.7 ± 5.4 [0-21.2]	7.7 ± 6.0 [1.4-17.7]	*2.7 ± 3.8 [0.6-24.3]*	1.7 ± 1.8 [0.6-7.2]	2.5 ± 2.1 [0.6-7]	6.3 ± 7.5 [1.7-24.3]
Insuline (mUI/l)	*6.6 ± 5.1 [1.5-23]*	5.2 ± 4.9 [1.5-23]	6.7 ± 4.1 [1.5-17]	11.0 ± 5.5 [3.6-21.1]	*9.3 ± 10.0 [1.5-58.2]*	5.7 ± 5.3 [1.5-22.6]	11.3 ± 13.2 [1.5-58.2]	15.5 ± 7.6 [3.3-28.1]
IGF-1 (µg/l)	*98.5 ± 46.4 [21.9-191]*	107.0 ± 47.9 [21.9-191]	91.8 ± 44.2 [22.8-186]	83.8 ± 39.3 [23-144]	*89.3 ± 43.4 [14.7-179]*	94.2 ± 40.0 [19.8-179]	80.2 ± 47.5 [14.7-170]	92.9 ± 41.7 [36-155]
Leptin (µg/l)	*5.7 ± 4.0 [0.7-19.3]*	3.9 ± 2.9 [0.7-12.5]	5.9 ± 3.3 [1.9-16.1]	11.8 ± 3.3 [7.9-19.3]	*6.2 ± 5.3 [1.3-29.4]*	3.9 ± 2.9 [1.3-14.7]	7.4 ± 6.4 [2.2-29.4]	10.9 ± 4.7 [5.6-19.3]
Adiponectin (mg/l)	*7.9 ± 4.7 [1.13-26.1]*	8.0 ± 4.2 [1.13-22.3]	7.5 ± 5.3 [1.13-26.1]	8.7 ± 4.9 [2.65-16.6]	*8.9 ± 5.9 [1.23-28.9]*	8.8 ± 5.1 [1.23-20.7]	8.8 ± 7.0 [1.41-28.9]	9.4 ± 5.4 [2.36-16.3]
Ratio leptin /adiponectin	*1.07 ± 1.15 [0.045-5.04]*	0.67 ± 0.58 [0.045-2.04]	1.34 ± 1.46 [0.173-5.04]	1.90 ± 1.20 [0.68-4.38]	*1.52 ± 3.20 [0.068-20.9]*	0.81 ± 1.33 [0.068-6.62]	2.45 ± 4.91 [0.122-20.9]	1.63 ± 1.02 [0.344-3.77]
Testosterone (nmol/l)	*0.872 ± 0.42 [0.16-2.5]*	0.81 ± 0.31 [0.27-1.5]	0.91 ± 0.51 [0.16-2.5]	0.96 ± 0.41 [0.7-1.9]	*0.85 ± 0.52 [0.19-2.8]*	0.72 ± 0.30 [0.21-1.9]	0.97 ± 0.70 [0.19-2.8]	0.99 ± 0.52 [0.7-2.2]
CA 15-3 (kU/l)	*21.0 ± 25.0 [5-150]*	26.3 ± 33.9 [6-150]	13.8 ± 7.4 [5-36]	22.6 ± 6.5 [12-31]	*18.4 ± 9.0 [3-38]*	17.9 ± 9.2 [3-37]	16.1 ± 8.2 [7-38]	25.3 ± 6.0 [14-32]

Diet parameters for food intake are expressed in raw value (gram / day) and in % of total energy intake. Body parameters are expressed in raw value (kilogram or liter) and in % of body mass. Plasma biological parameters are expressed in usual unit per liter.

PACThe Study design

Experimental design consisted of a multicenter parallel randomized prospective trial.
Patients were enrolled from March 2008 to October 2010. Inclusion criteria were:

- Invasive non-metastatic breast carcinoma.
- Less than 9 months after chemotherapy/radiotherapy completion.
- Complete remission.
- No contra-indication of physical activity: clinical examination and maximal exercise tests with determination of maximum oxygen consumption (VO_{2max}).
- 18.5 < BMI < 40 kg/m_2.
- No psychiatric illness.
- Informed signed consent.

Characteristics of the 2-week session performed in thermal centers:

- By group including 7–11 patients.
- Three SPA centers: Vichy, Le-Mont-Dore, Châtel-Guyon.
- Medical, nutritionist and psycho-oncologist consultations.
- Training courses lasted 13 full days when arrival and departure days in SPA center were excluded.
- Physical activity for 2 h daily, supervised by a physiotherapist:
  - Endurance activities: walking over a flat ground or pedal on a cycloergometer.
  - Physical exercises for both strength training and flexibility/stretching: upper and lower limbs and body were concerned.
  - Aquagymnastics.
- SPA cares consist of bath, shower and massage for half an hour per day. Personal hysiotherapy cares were also given if needed taking into account the entire body (usually physiotherapy performed in anticancer centers only focus on lymphoedema or pain/difficulties resulting from breast reconstruction).
- Aesthetic care.
- Dietary meals with adapted menus at the thermal resort, and dietary education. Caloric Intake was limited to 1700–2000 cal/day and dietary education was organized daily during SPA: it comprised cooking lessons related to the meals that patients had at lunch or supper. At every meal also, the chef visited the dining room and answered patients' questions about their menu and again the way to perform recipes.

Physical managements were described in detail by the PACThe protocol and physiotherapists of each SPA center agreed to respect carefully the general training programme. The quality of intervention was also the topic of two meetings organized by the main investigator at the SPA center, at the beginning and the end of each 2-week stay: besides the investigator, they gathered both SPA staff and patients' group. During the first reunion, participants and staff were introduced and goals and contents of the stay were precisely described. During the second, a debriefing with the same persons was performed in order to ensure no particular observation could indicate a protocol deviation, to check for patient satisfaction and to collect information that could suggest possible improvements. Besides standard oncological follow-up of the patients of both groups, personal consultations with a dietician were planned every 6 months until 3 years:

- To perform anthropometric measurements.
- To provide dietary advice with a dietician concerning cooking with less fat, based on a dietary inquiry about meals of previous week.
- To give encouragement for daily physical activity as defined by the American College of Sports Medicine (ACSM)/the American Heart Association (AHA) guidelines.

The French version of the SF36 questionnaire was used to evaluate the primary endpoint: QoL. Questionnaires were filled out before randomization at 6, 12, 18 months, and then at 2, 3 4 and 5 years.

Secondary endpoints were measured before randomization and at 6, 12, 18, 24 and 36 months for:

- Anxiety/depression (hospital anxiety and depression (HAD) questionnaire).
- Sleep (adapted from Leeds sleep evaluation questionnaire).
- Physical/sedentary activity scores (Ricci & Gagnon questionnaire for physical activity and questionnaire on sitting time).
- Diet consumption evaluation using a 72 h self-reported diet questionnaire.
- Body parameters: weight, waist circumference, body composition (impedanceometry).
- Biological parameters: measures were determined on half of the population at inclusion and one year later.
- Disease-free survival: data were collected over 7 years.

Sample size calculation was estimated from the primary endpoint which is the QoL. Previous investigations showed variation of SF36 QoL (measured using the mean of all scores by dimension) 6 months after such an intervention of 16% versus 4% in a control group (results not published). At 1 year, we expected a 10% differential increase in SF36 QoL score. With a type one error a = 0.05 and 95% power (b = 0.05), about 200 patients were necessary per arm. Randomization was balanced and stratified by menopausal status, BMI less or more than 25 kg/m2, and inclusion center.

This trial was performed in compliance with the Helsinki declaration. Protocol was approved by the AFSSAPS (French Agency for Sanitary Security of Health Products), the regional Ethics Committee (March 2008), and the French National Committee controlling personal computerized data (CNIL). This trial was registered in ClinicalTrials.gov with the n°NCT01563588.
